# Transformed Health Ecosystems—Challenges for Security, Privacy, and Trust

**DOI:** 10.3389/fmed.2022.827253

**Published:** 2022-03-25

**Authors:** Pekka Ruotsalainen, Bernd Blobel

**Affiliations:** ^1^Faculty of Information Technology and Communication Sciences (ITC), Tampere University, Tampere, Finland; ^2^Medical Faculty, University of Regensburg, Regensburg, Germany; ^3^First Faculty of Medicine, Charles University, Prague, Czechia; ^4^eHealth Competence Center Bavaria, Deggendorf Institute of Technology, Deggendorf, Germany

**Keywords:** ecosystem, security, privacy, trust, personal health information

## Abstract

A transformed health ecosystem is a multi-stakeholder coalition that collects, stores, and shares personal health information (PHI) for different purposes, such as for personalized care, prevention, health prediction, precise medicine, personal health management, and public health purposes. Those services are data driven, and a lot of PHI is needed not only from received care and treatments, but also from a person’s normal life. Collecting, processing, storing, and sharing of the huge amount of sensitive PHI in the ecosystem cause many security, privacy, and trust challenges to be solved. The authors have studied those challenges from different perspectives using existing literature and found that current security and privacy solutions are insufficient, and for the user it is difficult to know whom to trust, and how much. Furthermore, in today’s widely used privacy approaches, such as privacy as choice or control and belief or perception based trust does not work in digital health ecosystems. The authors state that it is necessary to redefine the way privacy and trust are understood in health, to develop new legislation to support new privacy and approaches, and to force the stakeholders of the health ecosystem to make their privacy and trust practices and features of their information systems available. The authors have also studied some candidate solutions for security, privacy, and trust to be used in future health ecosystems.

## Introduction

The ongoing health transformation aims not only at better understanding the causes of diseases and how drugs function inside the human body, but also at offering high quality health services for all at a lower level of cost. Thereby, innovative technologies and methodologies are deployed, such as digitalization, new mathematical tools for advanced modeling, artificial intelligence (AI), and machine learning (ML). In that context, a wide spectrum of personal health information (PHI) is collected that exceeds many times the content of current electronic health record (EHR). The intended outcome is the transformation of health and social care toward personalized, preventive, predictive, participatory, and precision medicine (5P Medicine) (1, 2). Other terms and definitions describing this development include Digital health, eHealth, and pHealth. All of them have many definitions. Digital health is an umbrella term covering concepts, such as mobile health (mHealth), health information technology, wearable devices, telehealth and telemedicine, and personalized medicine (3). It refers to the use of information and communications technologies (ICTs) in medicine and other health-related domains to manage illnesses and health risks, and to promote wellness (4). According to the WHO, eHealth transfers and exchanges health information between stakeholders and provides digitalized health services to support the delivery of health and the management of health systems (5). Moss et al. defined that “eHealth, or electronic health, refers to healthcare services provided with the support of information and communication technology” (6), and according to Eysenbach “e-health is an emerging field in the intersection of medical informatics, public health and business, referring to health services and information delivered or enhanced through the Internet and related technologies” (7). In this article the definition of Moss et al. is used. The core point in pHealth is personalized health. Ruotsalainen et al. have pointed that the pHealth user can be a patient, a customer, or a person managing own health/wellness, and pHealth collects a wide spectrum of PHI using sensors, and monitoring systems, and process that data using software applications and algorithms (2). pHealth services typically help a person to manage his/her own health, wellness, and lifestyle. Digital health, 5P Medicine, eHealth and pHealth, and mobile health have in common that they all are data-driven approaches. According to the National Institutes of Health (NIH), mobile health (mHealth) is “the use of mobile and wireless devices (cell phones, tablets, etc.) to improve health outcomes, health care services, and health research” (8).

The services of 5P medicine not only need data about care, treatments, and medication stored in the EHR, but also a wide spectrum of PHI, such as epigenetic data, personal health history, personally generated health information, the history of person’s health-related behaviors, and person’s individual characteristics. Personalized medicine refers to an approach that considers the patient’s genetic features and his/her preferences, beliefs, attitudes, and knowledge in social context (9). According to Prosperi et al., precision medicine is the “approach for disease treatment and prevention that takes into account individual variability in genes, environment, and lifestyle for each person” (10). Gorini et al. presented an even wider approach called 5P eHealth (11). In it, a patient is characterized not only as a biological and genetic entity, but also as a person with specific needs and values, habits and behaviors, hopes and fears, beliefs, personality, cognitive dispositions, health beliefs, social support networks, education, socioeconomic status, health literacy, and all the other life conditions and events (i.e., persons’ psychological characteristics). This means that the 5P eHealth creates the person’s full psychocognitive profile (11).

**FIGURE 1:**
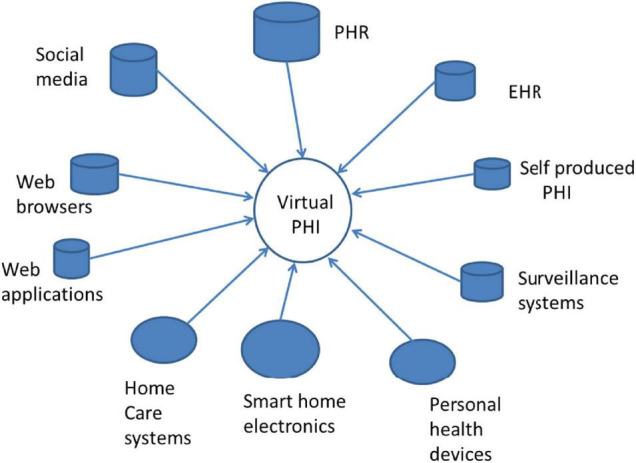
Typical sources of the personal health information (PHI).

To be successful, 5P approaches require PHI not only in the context of healthcare services delivery, but also before it, i.e., when we are “healthy.” This kind of PHI does not exist in any today’s EHR. Instead, it is necessary to collect PHI from many sources such as social media, Web browsers, personal health devices, and home care services (Figure 1). The combination of all those collected health data forms a “Virtual PHI” repository. This indicates a paradigm change, as the regulated EHR is not anymore in the center of the health ecosystem.

There is a big variety of sources of PHI, such as mobile phones, social networks, e-commerce applications, but also personal computer pedometers, smart health watches, smart wearables, and a smart toilet (12–14). Health and behavioral tracking takes increasingly place in public spaces, in cars, and in our work places (15). The collection of PHI using sensor technology is not limited to heart rate, body temperature, sleep patterns, or keyboard touching style. Even our emotions and behaviors can be monitored (16). Often, e-commerce services, surveillance systems, and commercial health applications collect, use, and store user related PHI, and monetize it.

Currently, eHealth, pHealth, and 5P medicine services are seldom stand-a-lone or end-to-end services. Instead, the ecosystems and the platform model are widely used. According to Iyawa et al., a digital ecosystem is “a network of digital communities consisting of interconnected, interrelated and interdependent digital species, including stakeholders, institutions, and digital devices situated in a digital environment, which interact as a functional unit and are linked together through actions, information and transaction flows” (17). The ecosystem metaphor requires that all stakeholders have a common goal, but in health ecosystems, this is not always clear. A precision medicine ecosystem links patients, providers, clinical laboratories, and researchers together for better care. An eHealth ecosystem adds government in the role of sponsor and regulator, and industry aiming at developing and selling medicine products (9). A platform is an ICT technical intermediate service that creates value for, and enables interaction between, customers and service providers. The platform operator orchestrates the services and communication between stakeholders in the ecosystem. Currently, a typical platform is Web-services in a Cloud. Vimarlund and Mettler have defined the eHealth ecosystem as a two-sided health service market platform that combines service consumers (persons or patients) and service providers, enabling them to profit from interactions by finding each other’s, and to reduce costs (18). Service examples include health portals, education, and self-care services.

Ecosystems built over platforms, clouds, and the Internet are widely used and, from economical, functional, and usability viewpoints, they can be successful in building blocks for pHealth, eHealth, and 5P services. Ecosystems can easily integrate stakeholders, such as service providers and service users and distributed information, which all can be located in different jurisdictional domains. Unfortunately, the architectural, functional, and technological features of ecosystems, especially when the large amount of sensitive PHI is communicated, processed, and stored, generate also meaningful security, privacy, and trust challenges. Some researchers have stated that in current digital information systems, it is almost impossible for the user to maintain privacy, today’s security solutions are ineffective, privacy is an illusion, and trust is only a belief (19–22).

From data subjects’ point of view, this kind of situation is unacceptable. It seems necessary to rethink the way privacy should be understood, what is its role in future information systems enabling 5P Medicine, eHealth, pHealth, and mHealth services can be, and how privacy can be implemented. In this paper, the authors study security, information privacy, and trust challenges existing in ecosystems supporting pHealth, eHealth, mHealth, and 5P services. Some answers to these problems will be presented in the next chapters.

## Privacy, Security, and Trust as Concepts

Security is a well-defined and standardized concept. According to the International Organisation for Standardisation (ISO), security implies the preservation of confidentiality, integrity, and availability of information (also authenticity, accountability, and reliability can be included) (23). Confidentiality is the property that information is not made available or disclosed to unauthorized individuals, entities, or processes, and data availability is the property that data are accessible and usable upon demand by an authorized entity.

Information privacy (aka privacy) and trust are fuzzy concepts with many definitions. At general level, privacy is a human right. Information privacy addresses the question what we like others or other information systems to know about us. Most common privacy approaches are privacy as right and ability to control (make choices), privacy as legal construct, privacy as contextual integrity, and finally risk based privacy (24, 25). Privacy is often understood as the exclusion of others. The control approach to privacy is linked to the self-determination and freedom to hide personal secrets, but also freedom from surveillance and tracking (25). The notice-and-choice-model for privacy (consent) is widely used in the European Union (EU) and the United States. It is based on the idea that data collectors have (moral or legal) responsibility to inform data subjects (DS) which data are collected (25) and how these are used, and that the DS can make rational and information-based decisions concerning which data they are willing to disclose, and to whom. Similarly, privacy as risk approach expects that the DS has the ability to make realistic risk assessment and then calculate expected benefits against the possible negative impacts of the data disclosure. Nissenbaum’s privacy as contextual integrity approach is based on the assumption that every context (e.g., healthcare domain) can have own contextual privacy rules which regulate the information flow inside the context and with other contexts (26). It presents a social theory of privacy by representing privacy rules as a common agreement and not as a personal right. A common misconception is to understand privacy as confidentiality (i.e., security). Confidentiality means that a data controller or processor has responsibility to guarantee that only authorized persons or entities can access data. In the privacy as right approach, the DS has right to define own personal rules (policies) regulating the processing of PHI. Privacy is also a legal construct in many countries. According to Sokolovska, specific laws protecting information privacy exist in 120 counties (27). In the EU, the General Data Protection Regulation (GDPR) offers to the data subjects (the EU-citizen) some rights to control the processing of personal information.

Behavioral privacy is a quite new concept. It is derived from the fact that service user’s online behaviors (e.g., lifestyle patterns) are increasingly sensed and recorded using sensors, surveillance systems, and computer browsers to predict the behavior of consumers, employees, and citizens (28). Using data mining and analytics, businesses organizations and governments are able to create detailed profiles of persons and use it in predictive analysis. Tracking people’s movements online is an invasion of privacy. Online behavioral tracking is even more serious because people are not aware who is tracking them, why, and how collected information will be used (29).

Trust is a social norm (25), which acts as the glue making our society function. Trust does not exist only between persons, but also between a human and organizations, computers, and technology. The way trust is understood is culture and context dependent. Human trust is also a personal trait. General trust is based on belief or disposition, i.e., it is a tendency to trust others without proof. There are many other approaches to trust, such as perceived trust, subjective probability-based trust, trust as risk, and willingness to trust (30). Our perception, previous experiences, other’s opinions, and proposals impact the trust formulation that is both a cognitive and an affective process inside our brain. Trust is also transformative: if we trust an organization, we often trust other similar organization (e.g., hospital). In digital information systems, such as the health ecosystem, the person has to trust in organizations, technology, and computational features of the system, as well as in communication and computer applications. Computational trust imitates the human trust creation process, and its goal is to calculate the level of trust in a context (31).

According to Lilien and Bhargava, privacy and trust can be a symbiotic or an adversarial relationship. Both require knowledge of other (30). When the trustee (e.g., a health service provider) makes information describing its privacy features available to the trustor (e.g., a service user), it gains trust. At the same time, the high level of trust indicates to the trustee that their PHI is processed fairly, and there is low or no need to require additional privacy safeguards.

## Security, Privacy, and Trust Challenges in Health Ecosystems

In this chapter, security, privacy, and trust challenges in health ecosystems are studied from different perspectives, such as ecosystem, data subject, privacy and trust models, privacy law, information architecture, and computation as well as from the 5P medicine viewpoint.

### Ecosystem’s Perspective

As mentioned earlier, the health ecosystem combines different kinds of stakeholders, such as the data subject (a person or patient), public and private healthcare service provider organizations and providers, researchers, and research organization, commercial vendors, such as tele-operators and Web service providers, platform managers, pharmaceutical organizations, or private organizations offering health and wellness management services. Some of the service providers have a physical location, but others are virtual organizations. The service provided is often non-tangible, e.g., they address just lifestyle and health management related information. Service providers and other stakeholders can have different business models (e.g., offering health services or monetization of PHI, and selling it) and security and privacy policies. They can also locate in different jurisdictions. This all makes it difficult for the service user to know which privacy and security rules apply, when and by whom PHI is used, and how to control data disclosure and secondary use of PHI. In the ecosystem, there exists meaningful power asymmetry between the DS at the one side and data collectors and processors at the other side. This makes it difficult to balance the DS’s privacy needs and data processors’ business needs (25). Furthermore, the collection of DS’s behavioral data as well as health tracking is a widely used practice in health ecosystems, and the DS has in real life no way to control it.

Additionally, a huge amount of PHI is collected by stakeholders to produce services for customers. According to Prosperi et al., PHI, such as omics data, information on medications, EHR data, transcriptions, behavioral, social, environmental and genetic data, shopping and bank information, the content of social media, data created by wearable devices, the content of school and employment records, income information, and social security records, can be collected for further use (10) (as shown in ref. Blobel et al. Transformation of health and social care systems—an interdisciplinary approach toward a foundational architecture, in this volume). This PHI forms a very sensitive Health Big Data record that raises concerns regarding global surveillance and possible misuse. Other challenges include how and where this data can be securely stored and made available for a long time, how information privacy can be guaranteed, who owns the data, and who can access at what granular level the data. A big challenge is how to recognize and prevent the possible misuse of PHI and prevent future social, psychological, and economical harm of possible secondary use and misuse of it.

The data ownership of PHI is certainly a difficult question because PHI used in the ecosystem is a combination of regulated healthcare data (e.g., the content of the EHR), self-produced health information, data collected in ecommerce and business relationships, and hidden collected behavioral data. According to Evans, the legal ownership of health data should be non-exclusive (32). In real life, many organizations (e.g., organizations offering social network services) see that the ownership of self-disclosed data belongs to them. Confusion over the ownership of PHI and conflicting opinions on privacy rules make it difficult to manage privacy in the ecosystem and to know what its overall level of privacy is.

Privacy management in today’s healthcare is based on the approach of well-defined context, where data flow through its borderline is strictly controlled following healthcare domain specific regulations. Unfortunately, this approach will not work in the health ecosystems because a part of PHI needed is located outside the healthcare domain, borderlines in the ecosystem are virtual and dynamic, and laws regulating information processing in commercial organizations vary. Furthermore, stakeholders collecting and using PHI in the health ecosystem often have different business models and privacy policies.

Successful service requires the linking of information collected from many separate information sources at different times (Figure 1). The linking requires the availability of DS’s unique identifier or pseudonym. In ecosystems, there is no guarantee that all data sources use the same identifier, i.e., different identifiers are often used. This fact that the DS requires opportunities to access own (identifiable) raw data rises concerns regarding privacy and possible re-identification (32).

### Data Subject’s View

A person needs privacy to overcome the lack of trust, but also to prevent others to have power over him or her. The insufficient level of privacy causes the loss of autonomy enabling increased behavioral, social, and political control, manipulation and discrimination from service providers and the government. Even though privacy is a human and constitutional right, it is often balanced against other’s benefit or business objectives in real life. Unfortunately, long-term negative side-effects (harm) are frequently not taken into account in this balancing because they often take place later, and harm is difficult to monetize. DS’s privacy needs can be also simply overridden by the service provider.

Before disclosing PHI to a stakeholder in the health ecosystem, the DS needs to know whom to trust, why and how much? Furthermore, the DS has to trust that necessary security and protection safeguards are in place, and all stakeholders in the ecosystem have fully implemented security and privacy requirements set by the laws of the DS’s home country. A meaningful challenge is that the DS seldom has necessary and reliable information for trust building and therefore for making informed decision how much PHI they are to disclose. Instead, belief-based trust or perceived trust are often expected by the service provider in real life (21), and the DS seldom has the possibility to define own privacy policies despite existing standards enabling such service (2, 22). Furthermore, the disclosure of PHI is in many cases not a free and a voluntary decision (25). Instead, the take-or-leave policy is widely used by the service providers, and behavioral data is invisibly collected using “mandatory” cookies.

The way human builds trust is a combined cognitive and affective process that can be, and is, widely manipulated. This fact together with the lack of reliable information of service provider’s and network’s security, privacy, and trust features leads to situations where the DS’s feeling or opinion about the trustworthiness of the ecosystem is the only measure. Unfortunately, there is no guarantee to what extent this feeling describes the actual trustworthiness of the ecosystem. A problem in Big Data environment is that the person’s information privacy is affected by other’s decisions, and DS’s consent is not sufficient to protect privacy (32).

The fact that precision medicine requires access not only to large-scale, detailed, and highly integrated PHI, but also to genetic information raises questions about who owns person’s genetic information, what results are returned, and to whom (9).

### Challenges With Privacy and Trust Models

Privacy as personal right and control is the most widely used approach in today’s information systems. It is based on the idea that a well-defined context of information processing exists and a rational evaluation of privacy risks and benefits is possible. Control rules, which can both reject and enable the processing of PHI, are typically expressed in the form of computer-understandable policies. This approach has many weaknesses, such as: in a pervasive environment, such as the health ecosystem, the control approach, and notice-and-choice (consent) is hardly to implement despite existing related standards. Rational decision-making frequently fails because of the limited rationality of humans (33), and the risk-based approach to privacy fails every day. Researchers have found that actual privacy risks are impossible to measure, and therefore the perceptions of opinion are widely used as proxy for actual risks. Unfortunately, perceptions are often only beliefs or based on other opinions. Privacy as contextual integrity approach fails also because contexts in ecosystems do not have clear boundaries, and inside the contexts, privacy rules are often defined by the stakeholder itself and cannot be defined by the DS.

Trust is a human trait that can be easily manipulated. General trust is a tendency to trust (belief) without any proof, i.e., it is unreliable. Trust as risk fails similar to the privacy as risk approach discussed earlier. Perceived trust is often only an opinion, and it is unreliable as well. Trust as subjective probability is problematic because the DS can hardly measure reliable probabilities. Computational trust based on own experiences and direct measurements is a promising approach, but its challenge is to get the reliable information of stakeholders’ and information systems’ trust features and behaviors.

### Regulatory Challenges

Current advanced privacy regulations, such as the EU GDPR or the California Consumer Privacy Act uses privacy as DS’s legal right and control approach. In this approach, the privacy right is a right to control (e.g., use consent) dissemination of personal data (25). This model that is widely used in the healthcare does not work in digital, distributed, and virtual ecosystem environment where regulations offer little protection (25). Furthermore, behavioral privacy is poorly or not at all protected. Current regulatory privacy models work in domains having clear boundaries and similar jurisdictional tradition. Unfortunately, this is not the case in health ecosystems, where many stakeholders other than the DS have legitimate interests in a person’s PHI (32). Laws, e.g., the EU GDPR, often give the data collector or processor the right to define the data it has legitimate interest in, to define the content of legitimate interest, and to use so called “mandatory cookies.” All those facts make it difficult for DS to control the use of own PHI in ecosystems. Health ecosystems running pHealth, eHealth, and 5P medicine are also Big Data environments, where informed consent is not capable to protect the DS against research-related privacy risks, and where cross-correlation among multiple datasets can enable re-identification (32). According to Evans, informed consent, giving the DS in real life situations only a take-it-or-leave-it right, is not adequate in the context of modern Big Data science and in precise medicine (32). Furthermore, in the context of genomics, consent does not work because it is nearly impossible to know the future uses of data at the time of collection (34).

The EU GDPR requires that organizations and entities which are actually in the control of health information should proactively use data protection principle [art. 5(2), art. 24], and assess, implement, and verify that data processing complies with the GDPR (art. 24) (35). Unfortunately, there is no legal obligation to explain the DS how this is done and which protection tools are implemented. Furthermore, laws do not enable the DS to know which data at granular level are collected, what privacy protection safeguards are in place, and which data are disclosed to other stakeholders in the ecosystem.

### Architectural, Security, and Computational Challenges

In health ecosystems that use a platform to orchestrate communication between stakeholders and applications and also to store the collected PHI, it is necessary for the DS and other stakeholders to trust in platform technology and in platform managers. Between organizations, the trust builder is typically a legally binding service level agreement (SLA), where content and penalties are defined in a negotiation process. In addition, external certificates are frequently used. In a health ecosystem, this is a challenging task caused by the large amount of public private and commercial stakeholders. Furthermore, it is more than challenging for the DS because they have limited or no power to negotiate an SLA.

In the ecosystem, there are many security challenges, such as a Denial of Service (DoS) attack that impacts the availability of services and data. An unauthorized node in the network (e.g., a sensor node) may also send false information. The platform manager can be untrusted and make administration errors and include wrong users. There can be software bugs, malware, and malicious insiders. As service users do not have access to the platforms’ internal operational details, the confidentiality and integrity of data can be at risk (36). Inside the platform, PHI is typically encrypted. This rises the problem of how to search encrypted data and how to manage securely the required encryption keys. Users of the ecosystem typically do not belong to one specific domain, but often to different jurisdictions. Different users need different access rights to PHI at different granularity level, using corresponding decryption keys. This makes authorization and key management a challenging task (36). Another challenge is how to guarantee long-term availability and integrity of PHI and how to proof data ownership during the whole retention time?

Cloud-based systems often use virtualization, i.e., multiple users run applications parallel on the same physical hardware. This generates security threats and privacy vulnerabilities to both the cloud infrastructure and cloud users (36).

### Challenges Linked to 5P Services

As discussed in previous chapters, personalized, preventive, predictive, participatory, and precision medicine services require a large amount of PHI, such as genetic information, clinical information extracted from patients EHR, and different kinds of PHI collected by non-regulated health service providers and commercial Web-sites. This raises privacy and data ownership concerns discussed in earlier chapters. Technologies used for the predictive and personalized health services include mathematical algorithms, modeling, AI, and ML. Heterogeneous and noise data from different environments, used in AI and ML, can produce biased and wrong results, and ML can generate results that are difficult to interpret by a human (37). The use of genetic information together with the content of EHR and/or PHI for profiling can also cause discrimination.

Research and commercial organizations offering AI and ML services are increasingly actors in the health ecosystem. This raises privacy and trust concerns especially in a situation where PHI without encryption is disclosed to them for personalized analysis and predictions, but increasingly also in the context of clinical studies. Commercial and research organizations are seldom certified for privacy, and their trust features can be unknown. Data anonymization does not help because identifiable information is needed for personalized services, and anonymization is insufficient to guarantee the unidentifiability of genetic data due to the existing auxiliary information.

Modern medical and health research is often multi-disciplinary and international. This raises trust concerns, because it is difficult to know who the authorized users of data are, how secure the information systems of participants are, and how privacy can be managed. Another challenge is to grant only necessary access rights to remote users, and to verify whether researchers asking for data access are legitimate and trusted.

Health Big Data and modeling enable to create a digital copy (Digital Twin) of human organs or even of the patient, and to use this copy for personalized medicine and disease prediction. The concept of Digital Twin raises privacy questions, such as who is the owner of person’s Digital Twin (e.g., DS, heath care provider, or somebody else), by whom Digital Twin can be used, and which are the rights of DS having Digital Twin (38).

## Privacy and Trust Solutions

As discussed in previous chapters, researchers have found that current security tools cannot guarantee privacy. Control-based privacy solution and belief-based trust do not work in health ecosystems (21, 39). To meet those challenges, researchers have developed new conceptual, organizational, and regulatory, but also information technology solutions. An overview of promising solutions is shown in Table 1. Some of those solutions propose only small modifications to the currently used privacy and trust approaches, but others are rather radical.

**TABLE 1:** Examples of new privacy and trust solution for health ecosystem.

Privacy focused solutions	Patient controlled EHR sharing	Use of cryptography	Computer understandable privacy policy
	Blockchain-based EHR repository	Blockchain- and smart-contract-based SLA	Privacy as control, and use of e-consent
	Mapping law and DS’s privacy needs	Privacy as regulatory property	Policy and ontology driven systems

Trust focused solutions	Privacy as trust, trust as fiduciary duty	Measurement of the level of computational trust	Collective agreement

Combination of privacy and trust	PHI as personal property and trust as fiducial duty, Blockchain based SLA		

Coiera et al. have developed an e-Consent mechanism to access PHI in electronic environment. This solution deploys the privacy as right and control approach and an e-Consent instead of traditional consent. In this solution, the e-Consent is a digital object that explains the specific conditions under which the PHI can be accessed, and by whom. According to Coiera, e-Consent can be general, general with special denials, or general denial with specific consents (40). A challenge in this approach is that it is difficult for the DS to manage granular and contextual e-consents, and therefore, this solution leads to very wide consents. Another related approach is addressed in the IEEE 7012 Project on Machine-Readable Privacy Terms the second author is member of Coiera and Clarke (41).

Another solution that is based on the patient’s right to control, but does not use consent, is the patient controlled health data sharing proposal. Here, the patient (or the person) dynamically controls the access to PHI stored in personally owned PHI repository, or to the content of regulated EHRs. Fatokun et al., for example, have developed an EHR system where the healthcare provider can search for patient’s data by requesting the patients’ agreement to access it. In this solution, the patient can manage the use and sharing of PHI and the content of the EHR. By using the Ethereum Blockchain platform and smart contract to guarantee security and non-repudiation, all patient data are stored on the peer-to-peer node ledger (42). Encryption is still needed for privacy, and the management of encryption keys can be challenging for the DS.

Researchers have developed many cryptographic solutions to protect the PHI’s integrity, availability, and confidentially. Data encryption is routinely used in cloud storages and during communication. In large data bases, encryption solution, such as differential privacy and K-anonymity are widely deployed to enable confidential data access and sharing. Homomorphic encryption seems to be the ultimate solution, but currently it supports only a few algorithms. Cryptography-based Blockchain technology has the power to guarantee the integrity and availability of data, but encryption is needed for confidentiality and privacy. Moreover, cryptographic technology is used for patient controlled data sharing. In a solution developed by Dubovitskaya et al., different hospitals are nodes in a permissioned Blockchain-based system aimed at EHR data sharing. Patients and doctors use a web-interface to initiate EHR sharing transactions. In this solution, original EHR data are stored outside a storage cloud, and a public key infrastructure based on encryption and digital signatures are used to enable secure storing and sharing of EHR data. The patients share their data using a Web service by specifying which data are shared to whom (43). In another solution developed by Chen et al., requested PHI is mapped to the privacy laws and requirements, to data users’ identity and to data owners’ disclose policy. Based on the results of mapping, decision to share or not to share data can be made. If needed, K-anonymity is used to secure the shared data (44). Cryptographic solutions can offer the high level of privacy, but the management of encryption keys is challenging as mentioned before already, and trust is only a strong disposition, or it should be created using other methods.

Security, privacy, and trust problems discussed earlier are caused by the complex, highly dynamic, and multi-disciplinary transformed health ecosystem. Those characteristics are not limited to the aforementioned security, privacy, and trust aspects or properties of, and perspective on, ecosystems. It of course also holds for designing, implementing, and managing the entire transformed health ecosystem itself. In the introductory paper of this volume, Blobel et al. noted that a more general system oriented view is needed, i.e., the challenge is to formally represent the specific aspects, intentions, and interests of all stakeholders (users) in their current and usually multiple contexts, to interrelate them and to integrate them properly in the business process to best meet the harmonized business objectives. A sustainable, future-proof approach to this challenge is the representation of the transformed health ecosystem as a system of systems by a system-oriented, architecture-centric, ontology-based, policy-driven model and framework, which has been meanwhile standardized in ISO 23903:2021 Interoperability and integration architecture—model and framework (45). This approach represents any system by its (knowledge) domains, i.e., user-specific and domain-specific perspectives and representation means (languages and ontologies), by generic granularity levels to allow correct and consistent interrelations, and finally by its evolution, e.g., a solution or software development process. The behavior of systems is ruled and controlled by domain-specific policies, which could be a process policy, a legal policy, a privacy policy including an individual privacy policy, but also moral or ethical principles and frameworks. As mentioned before, those different domains must use-case-specifically, currently and therefore dynamically represent using the corresponding domain ontologies (45–48). For the ontological representation of policies, ISO 22600:2014 should be used (49).

In addition, there are researchers who see that the current widely used privacy as right and control approach should be replaced by a new approach. Waldman has presented a privacy as trust approach, and Dobkin and Balkin as another approach, where trust is based on the regulated specific information fiduciary (25, 50, 51). These approaches require new legislation, and in real life it is difficult to know that the data collector/processor behave as required in the duty. Ritter et al. have proposed a regulation for data as property. In this approach, data ownership is clearly defined, but also at the same time property rules which define the right to own information (52). Natural persons and a legal entity can own data property, and only public data are understood as open data. Property also means that the data must be anonymized if a person does not accept the use of PHI. Also here, new law is needed, and encryption for privacy.

Since ownership right may not be sufficient against all privacy risks in digital environment and do not prevent against re-identification, Ruotsalainen et al. have proposed a model that combines the PHI as personal property approach and trust as regulated fiducial duty. In this solution, DS and data processor make a digitalized SLA using Blockchain smart contract technology. In this solution, the smart contract stores also trust duty requirements (21). A specific law for informational trust duty is needed, and the DS needs the information of trustee’s privacy and trust features before signing the smart contract.

Prosperi et al. have proposed a “health avatar” solution where avatar is a virtual representation of a person with all associated health information. The avatar captures and integrates health-related data, from genomics to omics, mobile, and wearable technology generated data, and environmental information (10). According to Prosperi, “Within the context of appropriate ethics bylaws and informed consents, health avatars could directly feed individual-level health information to multiple research projects.” In this innovation, the avatar is an active computer application that is programmed to collect data and make privacy decision according to DS’s will. A challenge is that data processors can regard avatars dangerous, and it is challenging for them to give restricted access control rights to the avatar. Evans has presented a radical approach to reject all traditional regulatory norms and replace them with collectively agreed norms (consumer-driven data common approach) (32). A weakness in this approach is that collectively accepted norms can be difficult in a heterogeneous group. Furthermore, norms provision and DS’s privacy needs can be conflicting.

There are other less radical proposals, such as offering the DS information to measure the level of privacy in the ecosystem. This approach can be used as the front end for different access control and data sharing solutions, such as the use of computer understandable privacy policies. The challenge is that the measurement of the actual level of privacy in a health ecosystem is a demanding task caused by the number of different stakeholders and many contextual factors impacting privacy (e.g., technology used, how security and privacy requirements are defined in laws, how standards are implemented in information systems, how stakeholders’ privacy policies and business models differ, how information is used, what is the sensitivity of data, and how the level of trustworthiness vary between stakeholders) (53). Another problem is that there is often the lack of reliable privacy and trust information in real life.

Distributed storage architecture is an architectural solution for the trustworthy disclosure and use of PHI. It splits the PHI into different blocks and stores them in different databases (or in blockchain ledgers), and the DS can grant separate access to each data block. This solution enables the detailed disclosure of extremely sensitive PHI, such as genomic data (54). In this solution, encryption is needed, and the management of granular encryption keys remains a challenge.

## Discussion and Conclusion

The ability to protect information privacy and high organizational trust has been for long a “*de facto*” requirement in healthcare. The ongoing health transformation toward personalized preventive, predictive, participative precision medicine, and healthcare challenges what PHI is collected, stored, used, and shared, and how privacy and trust are understood and created. Personalized and preventive services and new medical research need PHI that considerably exceeds the content of the today’s EHR. Digital measurement tools (e.g., sensors and monitoring devices) and communication technology have all together enabled the collection of personal related data almost on-line, supporting the aforementioned 5P medicine services. Furthermore, health and increasingly healthcare services are moved to ecosystems. At policy level, parallel to this transition, to gain economical, administrative, and social benefits, the general interest seems to move from strong protection to the balancing of information privacy and the free movement of health data (55). Furthermore, medical and health industry and e-commerce increasingly see PHI as “new oil” and commodity.

This development raises many privacy and trust challenges. Currently, it is nearly impossible for the health service user to guess, which privacy and trust principles and security and privacy solutions will best fulfill their privacy and trust needs, and at the same time to respond to the security, privacy, and trust challenges existing in the health ecosystem. In this paper, the authors have studied security, privacy, and trust challenges in health ecosystems from different viewpoints (ecosystem; data subject; regulatory, privacy, and trust models; architectural, security, and computation; and 5P Medicine) and recognized many issues to be solved. Novel privacy and trust solution prosed in the literature are also studied. Some of them are only enhancements to the current practice (e.g., e-consent), others rely on technology (use of cryptography), and some present a radical change (e.g., privacy as trust approach). All discussed proposals have their own limitations (Chapter 4), and none of them is widely accepted or used. Hence, there is much space for new innovations. The authors’ proposal for health ecosystems is the combination of privacy as personal property and trust as fiducial duty approaches in such a way that the duty to trust is created using legally binding smart contracts (56).

In any case, the authors state that for making pHealth, eHealth, and 5P Medicine successful, trustworthy, and secure, and therefore acceptable for people, it is necessary to redefine the way information privacy and trust in health ecosystems are currently understood and managed. To achieve this, widely accepted consensus, new laws, and political will are inevitable.

The redefinition of privacy and trust should be an international and multi-professional consensus, for example, under the guidance of the WHO. New regulations are also needed to enable the DS to evaluate (or calculate) the actual level of privacy and trust of the health ecosystem, and to force the ecosystem’s stakeholders to openly publish detailed privacy, security, and trust information concerning their information systems and processes. Since stakeholders in health ecosystem can locate in different jurisdictions, created laws (e.g., the law for specific fiducial duties) should be internationally accepted.

If the current, from the DS’s point of view, unsatisfactory situation will persist (i.e., nothing is done by regulators and policy makers and industry), the danger that our PHI will become commodity which is monetized comes true, and privacy and trust in health information system remains only a myth.

## Data Availability Statement

The original contributions presented in the study are included in the article/supplementary material, further inquiries can be directed to the corresponding author/s.

## Author Contributions

PR wrote the main part of this manuscript. The content and the title of this article were developed together by the authors. BB wrote part of the section “Privacy and Trust Solutions” and did a lot of the editing and fine tuning work necessary. Both authors contributed to the article and approved the submitted version.

## Conflict of Interest

The authors declare that the research was conducted in the absence of any commercial or financial relationships that could be construed as a potential conflict of interest.

## Publisher’s Note

All claims expressed in this article are solely those of the authors and do not necessarily represent those of their affiliated organizations, or those of the publisher, the editors and the reviewers. Any product that may be evaluated in this article, or claim that may be made by its manufacturer, is not guaranteed or endorsed by the publisher.
